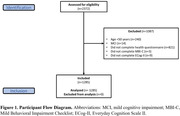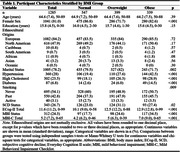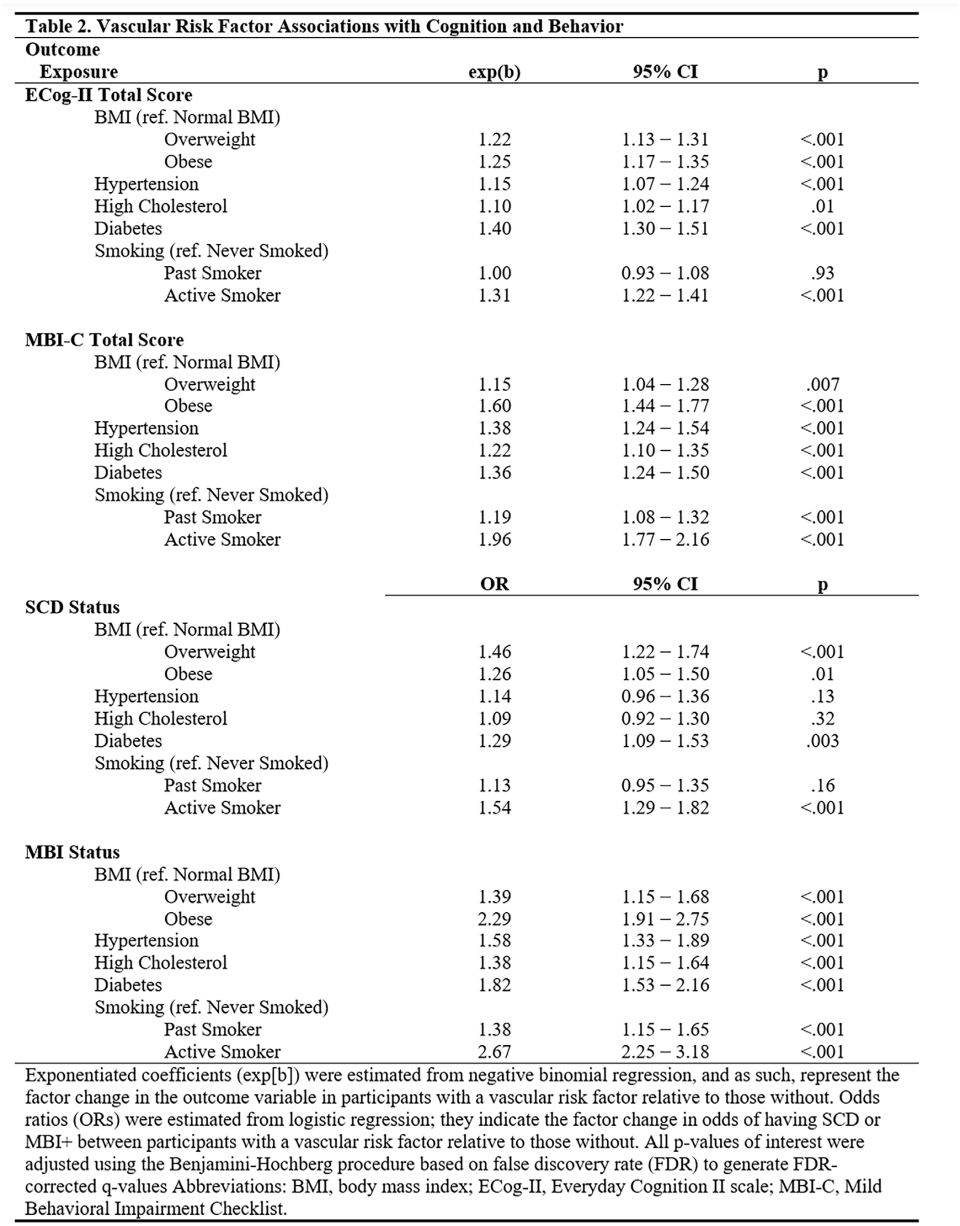# Vascular risk factors, subjective cognitive decline, and mild behavioral impairment: A CAN‐PROTECT study

**DOI:** 10.1002/alz.091470

**Published:** 2025-01-03

**Authors:** Dylan X. Guan, Aditya Aundhakar, Sarah Tomaszewski Farias, Eric E. Smith, Zahinoor Ismail

**Affiliations:** ^1^ Hotchkiss Brain Institute, University of Calgary, Calgary, AB Canada; ^2^ Department of Clinical Neurosciences, University of Calgary, Calgary, AB Canada; ^3^ University of California, Davis School of Medicine, Sacramento, CA USA; ^4^ Department of Clinical Neurosciences and Hotchkiss Brain Institute, University of Calgary, Calgary, AB Canada

## Abstract

**Background:**

Subjective cognitive decline (SCD) and mild behavioral impairment (MBI) identify older persons that are more likely to be at preclinical stages of Alzheimer’s disease (AD) than those without SCD and MBI. However, vascular co‐pathologies may also contribute to new onset and persistent cognitive and behavioral symptoms. We investigated vascular risk factor associations with SCD and MBI in older persons without mild cognitive impairment or dementia.

**Method:**

Data for 1285 participants from the Canadian Platform for Research Online to Investigate Health, Quality of Life, Cognition, Behaviour, Function, and Caregiving in Aging (CAN‐PROTECT) study were analyzed [Figure 1]. Vascular risk factors were body mass index (normal, overweight, obese), hypertension, high cholesterol, diabetes, and smoking (never, past, active). Outcomes were measured using the Everyday Cognition (ECog‐II) scale and MBI Checklist (MBI‐C). SCD was operationalized based on a score of ≥2 (i.e., consistently a little or much worse) on any ECog‐II item. MBI+ status was defined by MBI‐C total scores ≥8. Propensity scores were used to balance age, sex, years of education, marital status, and ethnocultural origin across exposure groups using inverse probability of treatment weighting. Weighted negative binomial and logistic regressions were used to model vascular risk factor (exposure) associations with ECog‐II and MBI‐C total scores, and SCD and MBI+ statuses, respectively.

**Result:**

Participant characteristics are summarized in Table 1. As shown in Table 2, all vascular risk factors assessed in the study were associated with higher ECog‐II total score, with the exception of past (i.e., not active), smoking behavior. Diabetes and active smoking showed the largest magnitudes of effect in relation to ECog‐II total scores. Active smoking and obesity showed the largest magnitudes of effect in relation to MBI‐C total scores. When assessing outcomes as categorical variables (i.e., SCD+ and MBI+), the same associations were found.

**Conclusion:**

Vascular risk factors were associated with poorer everyday cognition, more severe MBI symptoms, and greater odds for classification as SCD+ and MBI+ in a sample of older persons without objective cognitive impairment. These findings suggest potential vascular contributions to cognitive and behavioral markers traditionally linked to AD.